# The impact of perceived social support and coping on distress in a sample of Atlantic Canadian health professional students during COVID-19 compared to pre-COVID peers

**DOI:** 10.1186/s40359-023-01218-y

**Published:** 2023-06-01

**Authors:** Pamela Button, Laura Fallon, Ken Fowler

**Affiliations:** 1 Student Wellness and Counselling Centre, Memorial University of Newfoundland, St. John’s, NL Canada; 2 Department of Psychology, Memorial University, Memorial University of Newfoundland, St. John’s, NL Canada

**Keywords:** University, Mental health, Health professional, Social support, COVID, Resilience

## Abstract

**Purpose:**

Students pursuing higher education and health professional (HP) programs (e.g., nursing, pharmacy, social work, medicine) experience stressors including academic pressures, workload, developing professional competencies, professional socialization, the hidden curriculum, entering clinical practice and navigating relationships with colleagues. Such stress can have detrimental effects on HP students physical and psychological functioning and can adversely affect patient care. This study examined the role of perceived social support and resilience in predicting distress of Atlantic Canadian HP students during the COVID-19 pandemic and compared the findings to a pre-COVID population of age and sex matched Canadians.

**Method:**

Second year HP students (*N* = 93) completed a survey assessing distress, perceived social support, and resilience and open-ended questions on student awareness of supports and counselling available to them, their use/barriers to the services, and the impact of COVID-19 on their personal functioning. HP student responses were also compared with age and sex matched Canadian peers from data collected prior to COVID-19.

**Results:**

It was found that HP students reported moderate to severe psychological distress, and while they reported high levels of social support on a measure of perceived social support they also reported that the COVID-19 pandemic made them feel isolated and that they lacked social support. It was found that the sample of HP students reported significantly higher psychological distress than the mean scores of the age and sex matched sample of Canadian peers.

**Conclusions:**

These findings call for creation of more tailored interventions and supports for HP students.

## Introduction

Post-secondary students (PSS) experience significant stressors including leaving home, high expectations, demanding workload, changes in supports and social networks, and balancing new responsibilities. Recent research has found that these stressors can be harmful to their mental health and well-being and mental health problems of PSS have become more frequent and severe [1–4]. While some research has found that PSS do not have worse mental health than non-students of similar age and suggest that the perception of such may be related to other factors including increased rates of help-seeking behaviours, decreasing stigma, or increasing mental health literacy [5], a scoping review by Linden and Stuart has found that the majority of studies have found evidence of poor mental health in PSS, with some research suggesting students have significantly worse emotional health and higher distress when compared to the general population [3].

Along with the stressors universally experienced by PSS, students in health professional (HP) programs (e.g., nursing, pharmacy, social work, medicine) experience additional stressors such as enhanced workload and academic pressures, developing professional competencies, professional socialization, the hidden curriculum (i.e., factors associated with an academic environment beyond training), entering into a clinical/practice environment, caring for patients, patient mortality, and navigating new relationships with colleagues [6–9]. As a result of these unique stressors and the high demands placed, HP students tend to experience higher than average stress levels [10, 11]. The resulting stress can have an adverse impact on both the physical and psychological functioning of HP students, which can in turn affect the quality of care they provide to patients [12, 13].

Compared to the general population prevalence rate of about 11–13% for psychological distress, prevalence rates for HP students tend to be significantly higher; a systematic review reported that the prevalence rate of mental health issues in medical students ranges from 12 to 25% [14]. Similar findings from Maser and colleagues found that when medical students from across Canada were compared with PSS graduates from the general population, medical students reported significantly higher rates of psychological distress, mood disorders, and suicidal ideation [15]. In a study comparing them to an age-matched sample from the general population, medical students reported significantly higher scores on a psychological distress measure (18.9% vs. 5.3%) [6].While there is limited research on prevalence rates of psychological distress among other HP student groups one study found that 46.6% of surveyed female nursing students experienced distress [16] and another reported the prevalence rate of psychological distress among pharmacy students as 61.1% [17]. Moreover, the majority of students in most HP programs report being at least mildly stressed [3, 18].

Evidence suggests that elevated stress levels in HP students are related to increases in psychological distress and mental illness [13]. Perceived medical school stress has been shown to be a predictor of mental health problems [6, 15, 19]. Medical students have reported higher rates of anxiety, depression, substance use, and suicidal ideation than the general population [14, 19, 20]. In a survey of nursing students, chronic and transient stress was positively correlated with avoidance coping behaviours and negative self-esteem [12]. What is more, stress during HP programs can impact students even after they finish their schooling; for instance, stress during medical school can lead to impairments in psychological functioning during professional life, which can subsequently impact the quality of patient care [21].

Ongoing and prolonged stress can lead to burnout, which occurs when job strain leads to negative changes in an individual’s attitudes and behaviours. For a HP, these changes could relate to loss of concern for patients, irritability, detached manner, pessimism, blaming others, and lack of creativity; burnout can lead to physical problems including sleep disturbances, headaches, stomach problems, and fatigue, and in general can lead to a decline in work performance [10]. The potential for burnout is especially high in high-stress professions in which there is potential for emotional heaviness, demanding hours, and physical harm – a category into which most health professions would surely fit [22]. Research has shown that HP students tend to experience burnout in much the same way as their counterparts in the work force [10]. In medical students, burnout rates have been reported from 30 to 80% [23–25]. In a recent study, Roberts and colleagues reported that students in advanced practice nursing and physician’s assistant programs experience even higher levels of burnout than medical students [26].

It would be remiss to overlook the impact of the COVID-19 pandemic on HP student stress. A comprehensive review on the psychological impact of COVID-19 found that the pandemic has commonly induced psychological reactions such as pervasive anxiety, fear (particularly related to contracting COVID-19), frustration, boredom, and loneliness in the general public, all of which have been shown to negatively impact quality of life and well-being [27]. Recent research conducted in China, Spain, India, and the Philippines has demonstrated that 16–37% of the public experienced significant levels of psychological distress in response to the pandemic [28–32]. In Canada, self-perceived mental health has decreased since the onset of the pandemic, with 54% of Canadians reporting excellent or very good mental health in 2020, compared to 68% in 2018 [33].

Emerging research suggests that the global pandemic has contributed to the development of additional acute stress for HP students beyond the stressors they typically experience. Research on the psychological impact of COVID-19 revealed that, of all groups surveyed, students and HP experienced the highest levels of stress, anxiety, and depression during lockdown [34]. Given these findings and the continuing presence of the pandemic in our daily lives, participants in the current study provided their thoughts about the impact of COVID-19 on their experience in their HP programs. Our comparison group from the Canadian general public will serve as a pre-pandemic peer group to our sample, which represents a cohort of individuals approximately one year into the pandemic.

Fortunately, there are ways to mitigate the stress, and subsequent burnout felt by HP students including increasing HP students’ skills and education around coping. Research has been conducted on the strategies used by HP students to cope with stress: across HP disciplines, commonly cited positive coping strategies include seeking social support from family and friends, problem solving, taking time for oneself, and physical activity [9, 10, 12].

Developing resilience skills may also help HP students decrease stress, avoid burnout, and can contribute to better and safer patient care. Resilience involves being able to bounce back from and positively adapt to stressors, change, or negative circumstances [35, 36]. Across health professions, resilience has been cited as a critical skill for the promotion of health and wellness in HPs [37–40]. While research on the effects of resilience in HP students is limited, a study by Tempski and colleagues found an association between resilience levels and quality of life in medical students [41]. Moreover, a review of the literature that does exist on resilience in HP programs revealed that most research supported the need for enhancing resilience in HP education [42].

Furthermore, social support has been linked to positive effects on one’s physical health and well-being. Cassel proposed that social support buffers against the negative consequences of stress [43]. Social support has been shown to be negatively associated with depression and burnout, and positively associated with mental and physical health [44], and is associated with well-being across a variety of ages [45, 46]. Perceived social support refers to people’s beliefs about the supports that are available to them, whether they are positive or negative, and whether they meet their needs. It is thought that the perception of social support is even more important than the actual supports that exist [47]. For HP students, perceived social support has been shown to have a supportive impact on medical students’ experiences with stress and academic performance [22, 47, 48] and is positively associated with coping and mental health for nursing students [49, 50].

By further understanding the stress and distress experienced by HP students and protective factors that are present including perceived social support and resilience skills, we can better provide support education to help mitigate burnout before it reaches detrimental levels. If unable to cope adaptively and manage their own experiences of stress and distress constructively, it is likely that HP students will be less effective in helping those who face similar challenges [51]. While previous studies have examined HP student stress and coping, the current study will compare the role of social support and resilience in predicting distress in students from HP programs and comparing these findings to an age and sex matched group of Canadian peers. This work will help address the current gap in Canadian literature in terms of the prevalence of stress/distress, coping, and resilience among HP students as compared with peers in the general Canadian population and will provide a more comprehensive and in-depth understanding of the ways in which HP students are coping relative to peers across Canada.

### Objectives

To examine the role of perceived social support, coping, and resilience in predicting distress of an Atlantic Canadian HP students (during COVID-19 pandemic) and to compare to a general population of age and sex matched Canadians (pre-COVID-19 pandemic).

## Methods

### Study setting and participants

Health professional students in the second year of their programs of medicine, nursing (two programs), pharmacy, and social work were invited to participate in this study during the Spring 2021 academic year. There was a total of 261 students invited to participate, with 93 students completing the measures. Given the potential sampling frame of 261 students, the resulting response rate was 36%.

To explore the relative level of psychological distress and perceived social support in the HP student sample (during COVID-19 pandemic) to a general population (pre COVID-19 pandemic) a sample of age and sex matched community members were randomly extracted from the Public Use Microdata File of the Canadian Community Health Survey – Mental Health (CCHS-MH) 2012. The CCHS-MH contains data associated with Canadians aged 15 years and older who were residing in private residences in the 10 provinces at the time of the survey [52].

### Data Collection and analyses

Participants were recruited through the Faculties of Medicine and Nursing and the Schools of Pharmacy and Social Work at an Atlantic Canadian University. The researchers provided a representative from each program with an email invitation for 2nd year students to participate in the study, with a link to an online survey including informed consent, measures, and an invitation to enter an anonymous gift card draw. Four invitations were extended to students, each administered at weekly intervals over a one-month period. The instrument took 15 min to complete.

Brief demographic information was collected. Psychological distress was measured using the Kessler Psychological Distress Scale (K-10), a 10-item screening tool of psychological distress assessing mood, anxiety, and self-worth over the past month [53]. Participants rate each item on a scale of 1 (“none of the time”) to 5 (“all of the time”); scores are summed, with low scores (< 20) indicating being psychologically well and psychological distress categorized as mild (20–24), moderate (25–29), or severe (> 29); the optimal cut off score of 24 is indicated. This measure has been found to have strong psychometric properties, with good discrimination and internal consistency [52, 53]. Resilience was measured by the Brief Resilient Coping Scale (BCRS), a 4-item measure designed to assess the tendencies in which people cope with stress in a highly adaptive manner; the BCRS score interpretation ranges included low resilient copers (4–13 points), medium resilient copers (14–16 points), and high resilient copers (17–20 points) [36]. This measure has been found to have adequate internal consistency and test-retest reliability [36]. Perceived Social Support was measured using the Social Provisions Scale – Short Form [44, 54], a 10-item self-report measure used to assess five dimensions of social support across a variety of populations. Each question involves a four-point Likert scale (1 = “none of the time”; 4 = “all of the time”). The five dimensions of social support are attachment (i.e., emotional closeness); guidance (i.e., advice or information); reliable alliance (i.e., assurance that others can be relied on during times of stress); social integration (i.e., feeling of belonging to a group of friends); and reassurance of worth (i.e., recognition of one’s competence). The SPS-10 has been found to have excellent psychometric properties, with strong internal consistency and a predictive power similar to the original SPS-24. Each of the scales have demonstrated discriminant and construct validity, as well as internal reliability across populations [44, 52, 54].

Open-ended questions were included to explore student awareness of supports and counselling available to them, if they have sought such services and if not, reasons why they have not, and if there are any barriers to seeking services both on campus and in the community. Participants were also asked about the impact of COVID-19 on personal functioning.

### Statistical analyses

All analyses were conducted using SPSS version 28 for Windows. Independent sample t-tests were conducted to compare the HP students and the age and sex matched general population group on each of the measures of social support, distress, and resilience measures. A regression analyses was used to determine the predictors of distress in both the general population and HP student samples. Variables entered in the model included sex, age, and the five subscales of the SPS (attachment, guidance, reliable alliance, social integration, reassurance of worth). The alpha values (statistical significance value) are reported throughout, although *p < .05* was the standard value used throughout this research to denote statistical significance. Qualitative (open-ended) questions were posed to HP students and content analysis by frequency was used; this involved counting and categorizing the frequency of specific words, phrases, or themes that appeared in each of the open-ended question responses.

### Ethics approval

An ethics proposal to conduct research with the HP programs was reviewed and approved by Memorial University’s Interdisciplinary Committee on Ethics in Human Research.

## Results

A total of 93 HP students completed the survey (Social Work: *n* = 28; Medicine: *n* = 27, Nursing Students [Program 1]: *n* = 19; Nursing Students [Program 2]: *n* = 12; Pharmacy: n = 7). A breakdown of participants by demographic variables is shown in Table 1.

**Table 1:** Participant Demographic Variables (N = 93)

Demographic Variable	Percentage
Gender	
Male	17.2%
Female	81.7%
Non-binary/third gender	1.1%
Age	
<24	52.7%
25–29	33.3%
30–34	6.5%
35–39	1.1%
40–44	1.1%
Program of Study	
Medicine	29.0%
Nursing (P1)	12.9%
Nursing (P2)	20.4%
Pharmacy	7.5%
Social Work	30.1%
Marital Status	
Single	41.9%
Married	9.7%
Divorced/Separated	1.1%
Cohabitating	7.5%
Committed Relationship	39.8%
Children	
Yes	7.5%
No	91.4%
Prefer not to say	1.1%
Place of Upbringing	
Urban	52.7%
Rural	46.2%
Prefer not to say	1.1%

Mean scores on the K10 by program of study are shown in Table 2. Overall, participants reported a mean K10 score indicating moderate levels of psychological distress (*M* = 27.6) and exceeding the high score cut-off of 24. A total of 11.8% of participants reported scores indicating that they were psychologically well (< 20), 20.4% of participants reported a mild level of psychological distress (20–24), 26.9% reported moderate distress (25–29), and 40.9% reported severe psychological distress levels (> 29). Students from all programs reported mean scores that exceeded the high-score cut-off, with pharmacy students reporting the highest score (*M* = 28.7) and medical students reporting the lowest score (*M* = 24.9).

**Table 2:** Mean scores on Kessler Psychological Distress Scale (K10) items by program of study

*In the past 4 weeks, about how often did you feel…*	Overall (*N* = 93)	Medicine (*n* = 27)	Nursing (P1) (*n* = 12)	Nursing (P2) (*n* = 19)	Pharmacy (*n* = 7)	Social Work (*n* = 28)
Tired out for no good reason?	3.69 (1.01)	3.15 (1.10)	3.92 (0.90)	3.68 (1.00)	4.14 (0.69)	4.00 (0.86)
Nervous?	3.31 (0.92)	2.96 (1.02)	3.50 (0.52)	3.47 (0.91)	3.29 (1.25)	3.46 (0.84)
So nervous that nothing could calm you down?	2.43 (1.04)	2.15 (1.06)	2.33 (0.65)	2.58 (1.07)	2.43 (0.79)	2.64 (1.16)
Hopeless?	2.28 (1.04)	2.19 (1.08)	2.08 (0.67)	2.32 (1.25)	2.43 (0.79)	2.39 (1.06)
Restless or fidgety?	3.30 (1.07)	3.22 (1.05)	3.92 (1.00)	3.05 (1.03)	3.57 (0.98)	3.21 (1.13)
So restless you could not sit still?	2.61 (1.13)	2.48 (1.16)	2.83 (1.03)	2.47 (1.17)	3.00 (0.82)	2.64 (1.22)
Depressed?	2.67 (1.04)	2.26 (0.98)	2.83 (0.94)	2.84 (0.96)	2.29 (0.76)	2.96 (1.14)
That everything was an effort?	3.27 (1.08)	2.93 (1.17)	3.17 (1.03)	3.26 (1.10)	3.43 (1.27)	2.61 (0.88)
So sad that nothing would cheer you up?	2.10 (0.91)	1.81 (0.74)	2.00 (1.04)	2.11 (0.99)	2.00 (0.82)	2.43 (0.92)
Worthless?	1.98 (1.08)	1.78 (0.89)	1.92 (0.79)	2.16 (1.30)	2.14 (1.07)	2.04 (1.23)
**K10 TOTAL**	27.6* (6.93)	24.9* (6.85)	28.5* (6.23)	27.9* (6.92)	28.7* (5.44)	29.4* (7.27)

*Scores exceed the high score cut-off (i.e., 24) and indicate moderate levels of psychological distress

Mean scores on the BRCS by program of study are shown in Table 3. The mean total score (*M* = 14.4) indicated that HP students are medium resilient copers. Medical students reported the highest level of resilient coping (*M* = 14.9), while nursing students (Program 1) reported low resilient coping levels (*M* = 13.6).

**Table 3:** Mean scores on the Brief Resilient Coping Scale (BRCS) items by program of study

*Consider how well the following statements describe your behaviour and actions*	Overall (*N* = 93)	Medicine (*n* = 27)	Nursing (P1) (*n* = 12)	Nursing (P2) (*n* = 19)	Pharmacy (*n* = 7)	Social Work (*n* = 28)
I look for creative ways to alter difficult situations	3.55 (0.94)	4.48 (0.89)	3.75 (0.62)	3.00 (1.16)	3.71 (0.49)	3.86 (0.89)
Regardless of what happens to me, I believe I can control my reaction to it	3.51 (1.00)	3.67 (0.96)	3.42 (1.00)	3.79 (0.92)	2.86 (1.22)	3.36 (0.99)
I believe I can grow in positive ways to deal with difficult situations	4.00 (0.72)	4.11 (0.89)	4.00 (0.74)	3.74 (0.65)	4.00 (0.58)	4.07 (0.60)
I actively look for ways to replace the losses I encounter in life	3.32 (0.89)	3.59 (0.93)	3.08 (1.08)	3.11 (0.88)	3.43 (0.54)	3.29 (0.81)
**BRCS TOTAL**	14.4** (2.31)	14.9** (2.32)	14.3** (2.05)	13.6* (2.79)	14.0** (2.08)	14.6** (2.12)

* categorized as low resilient copers

** categorized as medium resilient copers

Mean scores on the SPS-SF by program of study can be found in Table 4. The mean score on the SPS-SF (M = 34.3) exceeded the cut-off score of 30, indicating that HP students reported a high level of perceived social support. The highest SPS-SF score was reported by pharmacy students (*M* = 36.1) and social work students had the lowest score (*M* = 33.3). With regards to the SPS-SF subscales (maximum score of 10), participants reported the highest score for reliable alliance (*M* = 7.19) and the lowest score for guidance (*M* = 6.44).

**Table 4:** Mean scores on the Social Provisions Scale – Short Form (SPS-SF) items by program of study

*Please respond to the following questions*	Overall (*N* = 92)	Medicine (n = 27)	Nursing (P1) (*n* = 12)	Nursing (P2) (*n* = 19)	Pharmacy (*n* = 7)	Social Work (*n* = 27)
There are people I can depend on to help me if I really need it	3.52 (0.60)	3.67 (0.56)	3.25 (0.87)	3.47 (0.61)	3.86 (0.38)	3.43 (0.50)
There are people who enjoy the same social activities I do	3.28 (0.65)	3.52 (0.70)	3.33 (0.65)	3.11 (0.74)	3.71 (0.49)	3.04 (0.43)
I have close relationships that provide me with a sense of emotional security and well-being	3.45 (0.63)	3.48 (0.70)	3.50 (0.52)	3.53 (0.70)	3.57 (0.54)	3.32 (0.61)
There is someone I could talk to about important decisions in my life	3.65 (0.52)	3.70 (0.54)	3.50 (0.52)	3.74 (0.45)	3.86 (0.38)	3.54 (0.58)
I have relationships where my competence and skill are recognized	3.24 (0.62)	3.37 (0.69)	3.25 (0.45)	3.21 (0.63)	3.14 (0.69)	3.15 (0.60)
There is a trustworthy person I could turn to for advice if I were having problems	3.55 (0.62)	3.52 (0.70)	3.42 (0.67)	3.58 (0.61)	3.86 (0.38)	3.54 (0.58)
I feel part of a group of people who share my attitudes and beliefs	3.16 (0.66)	3.19 (0.74)	3.08 (0.52)	3.16 (0.69)	3.43 (0.54)	3.11 (0.69)
I have a strong emotional bond with at least one other person	3.57 (0.65)	3.56 (0.85)	3.67 (0.49)	3.53 (0.70)	3.57 (0.54)	3.57 (0.50)
There are people who admire my talents and abilities	3.27 (0.68)	3.52 (0.70)	3.17 (0.72)	3.00 (0.58)	3.29 (0.49)	3.25 (0.70)
There are people I can count on in an emergency	3.62 (0.51)	3.67 (0.56)	3.58 (3.67)	3.63 (4.90)	3.86 (0.38)	3.54 (0.51)
***SPS Subscale 1: Attachment (Items 1 & 10)***	7.14 (1.01)	7.33 (1.04)	6.83 (1.27)	7.11 (1.05)	7.71 (0.49)	6.96 (0.88)
***SPS Subscale 2: Guidance (Items 2 & 7)***	6.44 (1.14)	6.70 (1.30)	6.42 (1.00)	6.26 (1.33)	7.14 (0.90)	6.14 (0.85)
***SPS Subscale 3: Social Integration (Items 3 & 8)***	7.02 (1.15)	7.04 (1.40)	7.17 (0.83)	7.05 (1.35)	7.14 (0.90)	6.89 (0.96)
***SPS Subscale 4: Reliable Alliance (Items 4 & 6)***	7.19 (1.08)	7.22 (1.19)	6.92 (1.08)	7.32 (1.00)	7.71 (0.49)	7.07 (1.12)
***SPS Subscale 5: Reassurance of Worth (Items 5 & 9)***	6.50 (1.13)	6.89 (1.22)	6.42 (1.00)	6.21 (1.08)	6.43 (1.13)	6.37 (1.11)
**SPS-SF TOTAL**	34.3* (4.45)	35.2* (5.03)	33.8* (3.67)	33.9* (4.90)	36.1* (2.27)	33.3* (4.20)

*Scores indicate a high level of social support

In open-ended questions participants provided information about barriers to counselling and/or support they had previously experienced (*N* = 37). The most common responses related to accessibility (*n* = 22), including issues with wait times, hours of operation, the length and number of sessions available, and lack of available information. The second most common type of barrier reported was financial (*n* = 14), relating to the cost of private counsellors and a lack of knowledge regarding costs associated with university-affiliated services. Other types of barriers included time constraints (*n* = 9), stigma (*n* = 8), access to appropriate supports (*n* = 6), and confidentiality concerns (*n* = 4).

Participants were asked to comment on the factors that would make them more likely to seek mental health services/support (*N* = 67). The most common responses related to increased accessibility (*n* = 31), specifically shorter wait times, more appointment availability, fewer restrictions, easier booking processes, increased flexibility, and walk-in clinics/outreach would make them more likely to seek support. Another commonly cited factor was lower cost (*n* = 15), which related to increased insurance coverage, financial assistance, and more clarity about costs associated with various supports. Participants also reported that they would be more likely to seek support if they experienced increased psychological distress (*n* = 13), if their comfort was enhanced during counselling sessions (*n* = 13), if they felt that their faculty/school supported the seeking of mental health supports (*n* = 10), if there was increased awareness (*n* = 8), or if they had more time to do so (*n* = 6).

Finally, participants were asked to comment specifically on how COVID-19 impacted their mental health and coping (*N* = 72). The most common response was that it made them feel isolated or as if they had a lack of social support (*n* = 45). This related to the inability to see family and friends, feelings of loneliness, and a lack of community and socialization among classmates. The next most common response was that the pandemic led to declining mental health (*n* = 33), including increases in anxiety, depression, and stress, sleep disturbances, and decreased motivation. Some participants also mentioned school-related difficulties (*n* = 21), decreased access to adaptive coping mechanisms (*n* = 13), and disrupted routines/lack of structure (*n* = 10).

### Matched sample comparison

Independent samples t-tests were conducted to compare mean scores on the K10 and SPS (overall and subscales) in the HP student sample to those in a matched sample of the general population. The mean score on the K10 was significantly higher in the student sample than the general public sample, *t*(178) = -12.86, *p* < .001, *d* = 1.92, indicating that the HP students were significantly more distressed than the general population. The overall SPS score was significantly lower in the student sample than in the general population, *t*(177) = 4.52, *p* < .001, *d* = 0.68, which suggests that the general population sample experiences higher levels of social support than the student sample. Mean scores were significantly lower in the student sample compared to the general population for each of the five subscales of the SPS: attachment, *t*(178) = 2.67, *p* = .008, *d* = 0.40; guidance; *t*(178) = 6.06, *p* < .001, *d* = 0.90; reliable alliance, *t*(178) = 2.03, *p* = .044, *d* = 0.30; social integration, *t*(178) = 3.60, *p* < .001, *d* = 0.54; reassurance of worth, *t*(177) = 4.37, *p* < .001, *d* = 0.65.

Regression analyses helped determine predictors of distress in both the general population and HP student samples. Variables entered into the model included sex, age, and the five subscales of the SPS (attachment, guidance, reliable alliance, social integration, reassurance of worth). For the HP student sample, age and sex accounted for a significant proportion of the variance (*R*^*2*^ = 0.056) on the K10 total score, *F*(2,84) = 3.57, *p* = .033. All five of the SPS subscales also accounted for a significant proportion of variance (*R*^*2*^ = 0.304) on K10 total scores, *F*(7,79) = 4.92, *p* < .001. For the general population matched sample, the five SPS subscales accounted for a significant proportion of the variance (*R*^*2*^ = 0.294) on the K10 total score, *F*(7,79) = 4.70, *p* < .001.

## Discussion

This study explored the role of perceived social support and coping in predicting distress of HP students at an Atlantic Canadian University during the COVID-19 pandemic and compared the results to a general population of age and sex matched Canadians (pre Covid-19 pandemic). Results found that 2nd year HP students (*n* = 93) reported moderate to severe psychological distress, while also being medium resilient copers. While in the general population age and sex matched group, all five types of social support were found to be a significant predictor of distress, for the student in HP programs age, sex, and social support predicted distress. While students reported they felt they had high levels of social support on the Social Provisions Scale, on open-ended questions 72 students indicated that COVID-19 impacted their mental health and 45 of those students indicated that the pandemic made them feel isolated or as if they had a lack of social support. Moreover, responding HP students in this study reported significantly higher psychological distress than the mean scores of age and sex-matched CCHS-MH control samples.

### Distress

In the current study, 27% of HP students reported moderate distress and 41% reported severe psychological distress. While all HP groups reported psychological distress, students in medicine reported the lowest distress scores. Nearly 70% of the HP students indicated that they were experiencing distressed mood, heightened anxiety, and affected self-worth over the past month. Furthermore, the HP student sample was significantly more distressed than those in the general Canadian matched sample. Research has found that HP students reported experiencing higher levels of psychological distress during the pandemic. These findings are similar to those by Lyons and colleagues who found that 37% of Australian medical students reported they were moderately distressed and 26% reported high to severe distress [11].

### Social Support

An impact of COVID-19 that continues to emerge from the literature is the impact that isolation has had on students, in part due to quarantine [11, 55]. Lyons and colleagues found that in medical students the main negative impacts of COVID-19 have been on social connectedness, while positive impacts included family relationships [11]. In the present study, HP students indicated that they had high levels of perceived social support on the Social Provisions Scale, with the highest score reported for reliable alliance and the lowest score for guidance. Despite reporting an overall high level of social support by HP students, when compared to a sample of the general population it was found that the perceived social support of HP students during the COVID-19 pandemic was significantly lower than matched peers in the general population. Moreover, HP students reported significantly lower social support than the general population in all five dimensions of the SPS. This suggests that while HP students continued to feel that they were supported by their social networks, COVID-19 might have had an impact on the degree to which they felt connected.

**Distress and social support**. For HP students, sex, age, and social support were found to be significant predictors of psychological distress. In the general population (CCHS), all five types of social support were found to be significant predictors of distress, while sex and age were not found to be significant predictors in this population. Research on post-secondary students found that negative mental health outcomes and distress were a consequence of the pandemic [56], while others have found that younger adults may be at an increased risk for distress over COVID-19 than older adults [32, 57]. Students in HP programs can vary on the factors including age, years of academic and/or work experience, interpersonal supports, and level of autonomy, and undergraduate and graduate students can differ in their learning strategies, coping, self-motivation with those with more academic experience were better prepared to complete the emergency online education [58, 59]. To examine the potential impact of these factors on varied HP programs and variations of these variables, a larger scale study must be conducted.

### Resilience

Resilience is “a dynamic process encompassing positive adaptation within the context of significant adversity” [60], while Masten [35] defines resilience as an adaptational process and can therefore be learned. The current study measures resilience from the perspective of a skill that can be developed, a coping behaviour that can promote positive adaptation during times of adversity and challenge [36]. In the present study, a brief measure of resilience was used to determine the resilience of the HP sample. Overall, the HP students were medium resilient copers, with those in medicine having the highest reported levels of resilient coping and nursing students having the lowest. While past research has focused on resilience in medical students, these findings highlight the importance of understanding resilience in different HP students. The current research demonstrates an opportunity to build on what is strong and enhance HP student resilience and provides an opportunity for those working with these students to provide information, support, and education on developing resilience and adaptive coping strategies throughout their education.

### HP students and COVID-19

While research about the impact of COVID-19 continues to emerge, studies have found that COVID-19 has impacted post-secondary students and HP students in a variety of ways. Toth, LaBarre, and Murray found that COVID-19 impacted social work students on their field internships both mentally and emotionally, noting that many students reported negative impacts on their mental health [61]. Lyons and colleagues found that in Australian medical students, COVID-19 most negatively impacted their feelings of social connectedness, studies, and stress levels, while Wasil and colleagues found that in graduate and professional students, COVID-19 most impacted their productivity/work-related stressors, health concerns, and emotional problems [11, 62].

During the data collection stage for this research (April 2021), the COVID-19 pandemic measures had been ongoing for just over 1 year. Students were abiding by sheltering in place and public health recommendations during this time, participating in their academics through remote and online learning. When asked about the ways in which COVID-19 had impacted their mental health, students indicated that they felt isolated or lacked social support, including inability to see family or friends, feelings of loneliness and a lack of community or socialization among their classmates and peers. This is interesting when taken into consideration with their perceived social support, which was rated as high. The next most common response was the deleterious impact this had on their mental health, which is consistent with the near 70% of students who indicated that they experienced moderate to severe psychological distress. Finally, some students reported school related difficulties, decreased access to adaptive coping mechanisms, disrupted routines/lack of structure, interpersonal conflict, and financial stress.

In open-ended responses, barriers to accessing counselling or more formal supports included accessibility of services; financial barriers; time constraints; stigma; access to appropriate supports; and confidentiality concerns. This mirrors findings by Bartlett and Fowler, in which medical students were reluctant to seek help formally due to similar reasons, noting that these students tended to seek informal supports while highlighting the need for improved access to confidential care [6].

In terms of identifying factors that would make them more likely to seek mental health services/support, students identified increasing accessibility and removing the barriers to service. Students highlighted solutions to the barriers including increasing insurance coverage and/or financial assistance and better information about what supports are available to them. Students also reported that they would be more likely to seek mental health supports if they felt their faculty/school supported them [6].

Through providing HP students the opportunity to openly respond to questions on the impact COVID-19 and its impact on their mental health/barriers to seeking support, the pandemic has highlighted areas of strengths as well as gaps within student services and supports, including formal and informal supports. This provides an opportunity to those involved in the HP programs and the institution to gain better understanding of what their students need and how to best support them.

### Limitations and future directions

This is a correlational study, and as such we cannot determine the cause of student distress, while the cross-sectional design of the study limits the generalizability and interpretability of the findings. As such, causal inferences of HP student distress, resilience, social support is not supported. While this study was with 2nd year HP students at one institution and the results have limited generalizability, the findings help to identify key factors for future study. Furthermore, the findings of this study likely have limited generalizability related to the COVID-19 pandemic. The pandemic provides and ongoing unique opportunity to study the impact of a global pandemic, safety measures, and interventions including remote learning. This study was conducted during one time period during the pandemic and as such the findings in this study may not be representative of what another time period might look like.

It would be remiss to not acknowledge that the use of HP student data and age and sex matched sample (non-COVID time) did not account for other variables that might differ between these two groups. Those enrolled in HP programs may differ from the general population in a variety of factors including social factors such as socio-economic status, education levels, employment status, wealth, and privilege, to name a few. Furthermore, given the scope of this study there might also be differences between university students and those in HP programs; each of these considerations would be beneficial to explore in future research. Data collection was in the form of self-report measures, and as such the students who were willing and able to respond may have been experiencing differing levels of stress than other students, and social desirability may have also played a role.

Future research on HP student distress, resilience, and coping across a wider array of HP programs and across each year of academic programming would help us better understand the ways in which our future HPs cope and respond to unique stressors. A longitudinal study design would help us understand the ways HP students cope throughout their training and into early career. These results will help to inform the development of programs and services to help HP students cope with stress/distress and foster resilience and flourishing and will allow mental health services at the university and within each program’s faculty/school to provide services to help the future providers of health care in our province. As post-secondary institutions continue to navigate how to best offer student services and supports to students, ongoing evaluations of programs and understanding of what students’ need is paramount; ongoing program development and evaluation will aid in this process.

## Data Availability

All data related to the health professional students is freely available upon request to the corresponding author. The information and data related to the Canadian Community Health Survey can be found via Statistics Canada (https://www150.statcan.gc.ca/n1/en/catalogue/82-617-X).

## Declarations

### Author’s information

Dr. Pamela Button is an Assistant Professor and Registered Clinical Psychologist at the Student Wellness and Counselling Centre at Memorial University of Newfoundland.
